# pandasGWAS: a Python package for easy retrieval of GWAS catalog data

**DOI:** 10.1186/s12864-023-09340-2

**Published:** 2023-05-04

**Authors:** Tianze Cao, Anshui Li, Yuexia Huang

**Affiliations:** 1 School of Mathematics, Hangzhou Normal University, Hangzhou, 311121 China; 2 Department of Statistics, Shaoxing University, Shaoxing, 312000 China

**Keywords:** Database, Repository, RESTful, Python, GWAS, Pandas

## Abstract

**Background:**

Since the NHGRI-EBI Catalog of human genome-wide association studies was established by NHGRI in 2008, research on it has attracted more and more researchers as the amount of data has grown rapidly. Easy-to-use, open-source, general-purpose programs for accessing the NHGRI-EBI Catalog of human genome-wide association studies are in great demand for current Python data analysis pipeline.

**Results:**

In this work we present pandasGWAS, a Python package that provides programmatic access to the NHGRI-EBI Catalog of human genome-wide association studies. Instead of downloading all data locally, pandasGWAS queries data based on input criteria and handles paginated data gracefully. The data is then transformed into multiple associated pandas.DataFrame objects according to its hierarchical relationships, which makes it easy to integrate into current Python-based data analysis toolkits.

**Conclusions:**

pandasGWAS is an open-source Python package that provides the first Python client interface to the GWAS Catalog REST API. Compared with existing tools, the data structure of pandasGWAS is more consistent with the design specification of GWAS Catalog REST API, and provides many easy-to-use mathematical symbol operations.

## Background

The GWAS Catalog was founded by the NHGRI in 2008, which is a consistent, searchable, visualized and freely available database of all published genome-wide association studies [1]. Currently, there are three ways to access this data: (i) via the graphical search interface supported by official website, (ii) via downloading the offline data dump provided by the official website, (iii) via GWAS Catalog REST API hosted by official website. The first way is the most friendly to beginners, and can obtain the latest data, but it can only be operated manually, which is not convenient for automation based on programming. The second method can obtain all data locally, but cannot guarantee that the data is up-to-date at the time of research. The third method combines the advantages of the previous methods, but the steps of acquiring and parsing the data are tedious. Firstly, there are many URL parameters for requesting data, and beginners must read the documentation deeply to understand how to assemble the correct parameters. Secondly, the structure of the response data is also complex. Based on different request parameters, response will be in normal JSON format or JSON + HAL format [2]. At the same time, the format of data may also be paginated or not, or it may be in the form of Array or not.

## Implementation

### Retrieving data from server

**Fig. 1:**
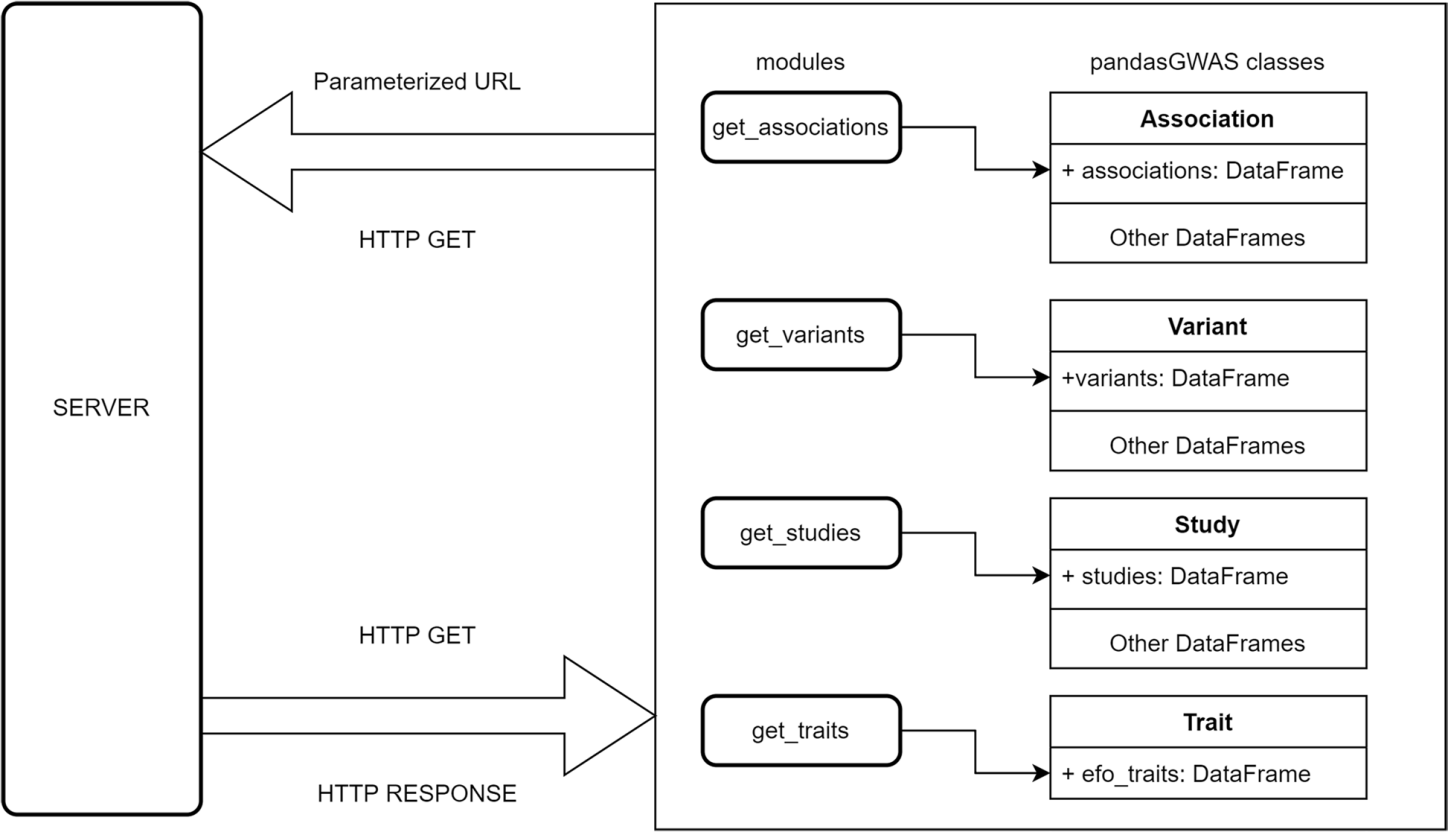
Architecture of pandasGWAS

pandasGWAS allows programmatic access to the GWAS Catalog data which leverages GWAS Catalog REST API [3]. HTTP response of GWAS Catalog REST API are categorized into Study, Association, Variant and EFO Trait. pandasGWAS provides various functions for the above 4 data types in the module get_associations, get_variants, get_traits, and get_studies, respectively. pandasGWAS assembles the requested URL based on the specific called function and the parameters passed in. If the raw data of response is in the form of JSON + HAL, pandasGWAS will automatically extract valid data from the "_embedded" property. If the data is paginated, pandasGWAS will in turn request data from other pages and aggregate all the data. For friendly interactive features, pandasGWAS uses the progressbar2 module to visualize this progress [4]. The processed response data is converted into an instance of the pandasGWAS custom class based on the called function (Fig. 1).

### Convenient set operations

In the module set_operation, pandasGWAS provides a variety of set operation methods for analysis between objects of the same type: bind(), union(), intersect(), set_diff(), set_xor() and set_equal(). pandasGWAS also supports set operations based on mathematical symbol operations: + (bind), &(intersect), -(set_diff), ^(set_xor), |(union), =  = (set_equal).

### Helper functions for accessing web links

In the module Browser, pandasGWAS provides a set of helper functions for accessing web links, such as PubMed(open_in_pubmed()), dbSNP(open_in_dbsnp()), GTEx project(open_in_gtex()) and the GWAS Catalog Web interface itself(open_study_in_gwas_catalog(), open_variant_in_gwas_catalog(), open_trait_in_gwas_catalog(), open_gene_in_gwas_catalog(), open_region_in_gwas_catalog() and open_publication_in_gwas_catalog()).

### Class structure of data entities

**Fig. 2:**
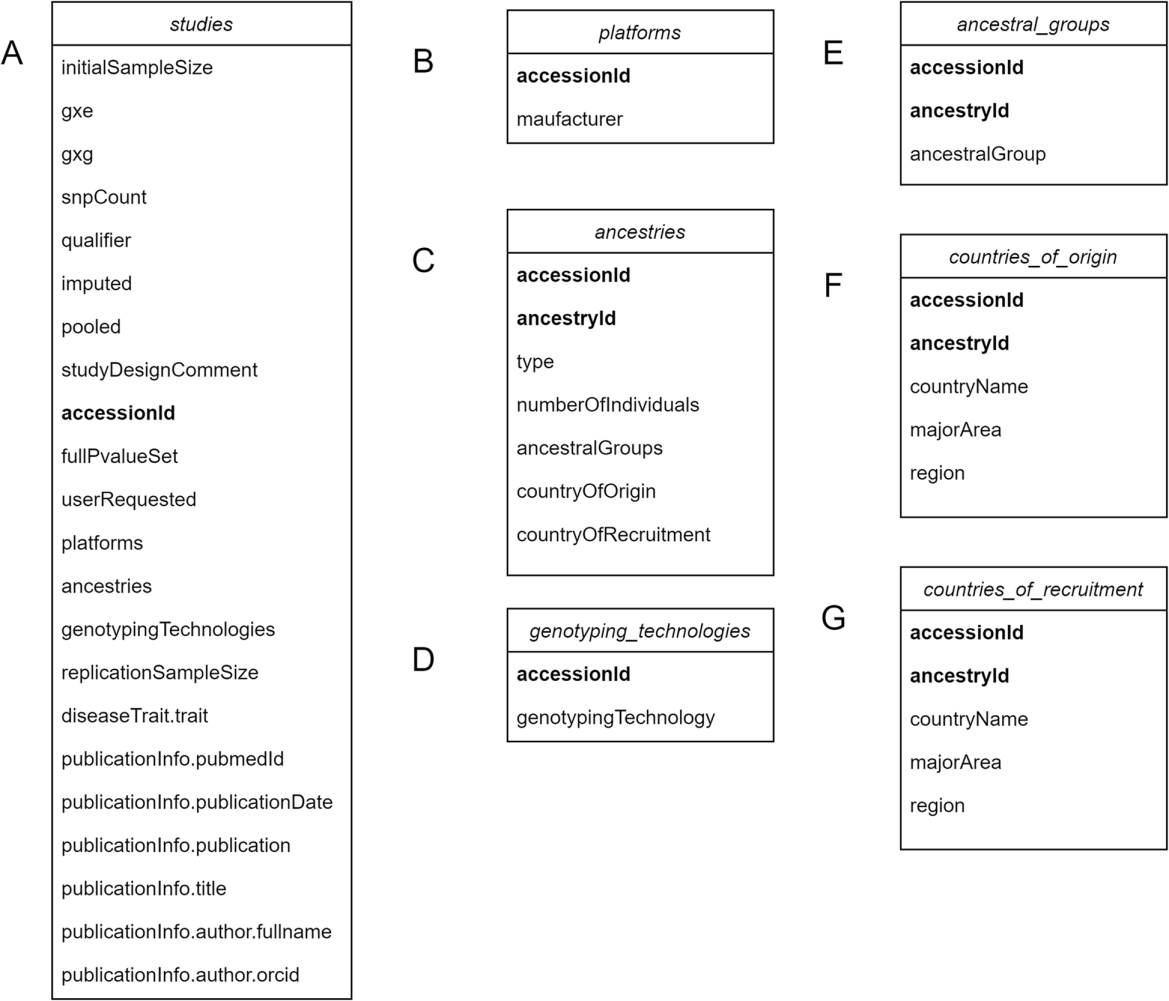
Class structure of Study. **a** columns of the property studies in class Study; **b** columns of the property platforms in class Study; **c** columns of the property ancestries in class Study; **d** columns of the property genotyping_technologies in class Study; **e** columns of the property ancestral_groups in class Study; **f** columns of the property country_of_origin in class Study; **g** columns of the property country_of_recruitment in class Study

**Fig. 3:**
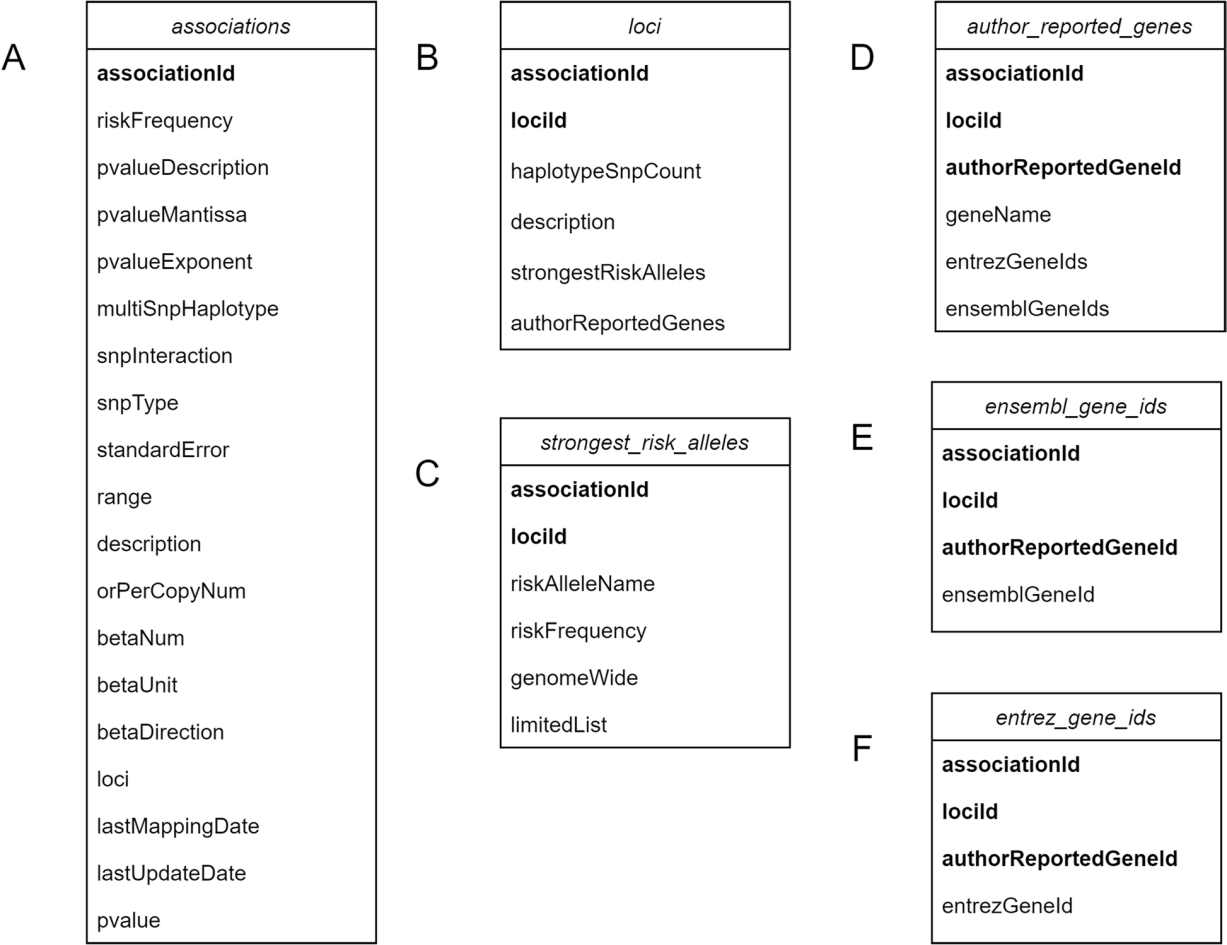
Class structure of Association. **a** columns of the property associations in class Association; **b** columns of the property loci in class Association; **c** columns of the property strongest_risk_alleles in class Association; **d** columns of the property author_reported_genes in class Association; **e** columns of the property ensembl_gene_ids in class Association; **f** columns of the property entrez_gene_ids in class Association

**Fig. 4:**
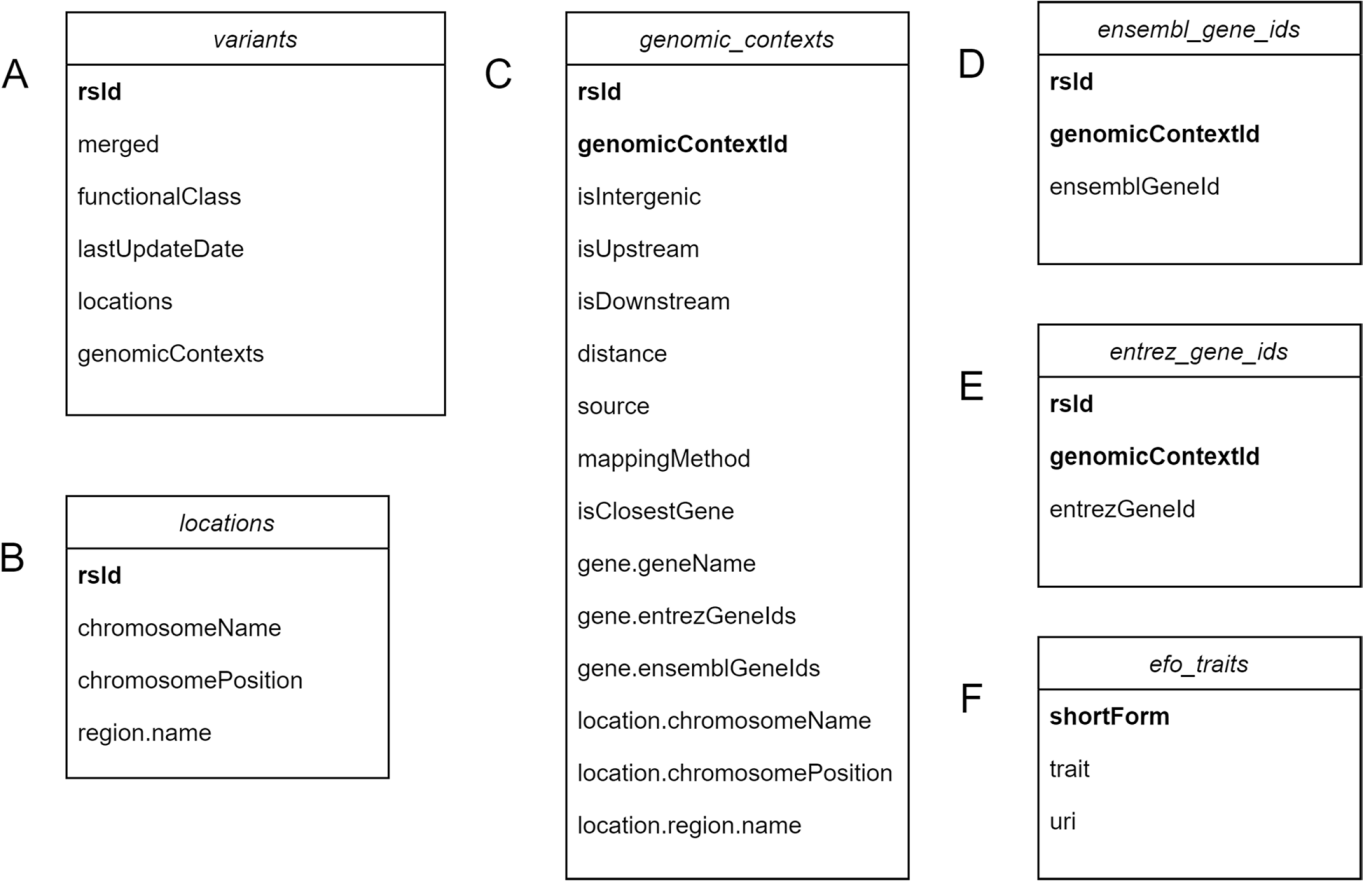
Class structure of Variant and Trait. **a** columns of the property variants in class Variant; **b** columns of the property locations in class Variant; **c** columns of the property genomic_contexts in class Variant; **d** columns of the property ensembl_gene_ids in class Variant; **e** columns of the property entrez_gene_ids in class Variant; **f** columns of the property efo_traits in class Trait

The class Study contains 7 properties: studies, platforms, ancestries, genotyping_technologies, ancestral_groups, country_of_origin and country_of _recruitment. The types of these properties are pandas.DataFrame [5]. When the processed data is passed into constructor of Study, constructor parses data into the property studies of which columns correspond one-to-one with properties of Study in GWAS Catalog REST API. The column accessionId is an identifier in the Study, which can be used to find a unique Study on the official website. Because platforms, ancestries, and genotypingTechnologies listed in studies are of type Array, they are flattened and assigned to properties with the same name to facilitate future data analysis. The value in the corresponding column accessionId is also assigned to the property platforms, which acts as a foreign key of relational database between the property platforms and the property studies, and also applies to the property ancestries and the property genotyping_technologies. Based on the same design principle, pandasGWAS creates the column ancestryId as the primary key of property ancestries, and extracts the corresponding values and assigns them to properties ancestral_groups, countries_of_origin and countries_of_recruitment respectively (Fig. 2). The properties of Classes Association (Fig. 3), Variant (Fig. 4A-E) and Trait (Fig. 4F) are designed with the same philosophy as the Class Study.

## Results and discussion

### Example 1: a real world use case

To demonstrate the utility of pandasGWAS, we use the work of Light et al. as an example [6]. In this work, the authors started by selecting variants previously reported in the GWAS Catalog for autoimmune disease. It can be easily implemented using pandasGWAS. Firstly, we load the required modules in the Python console.

 >  >  > from pandasgwas.get_studies import get_studies

 >  >  > from pandasgwas.Browser import open_in_pubmed

 >  >  > from pandasgwas.get_associations import get_associations

Then we can get studies in the GWAS Catalog by autoimmune disease.

 >  >  > my_studies = get_studies(efo_trait = 'autoimmune disease')

We can use the function len () to confirm how many studies were retrieved.

 >  >  > len(my_studies)

We can know the Study identifier easily.

 >  >  > my_studies.studies ['accessionId']

To browse related study directly on PubMed, we can use the helper function open_in_pubmed ().

 >  >  > my_studies.studies ['publicationInfo.pubmedId'].apply(lambda x:open_in_pubmed(x))

To get the variants previously associated with autoimmune disease.

 >  >  > my_associations = get_associations(efo_trait = 'autoimmune disease')

To filter associations by *P* value < 1 × 10^−6^.

 >  >  > association_ids = my_associations.associations [my_associations.associations ['pvalue'] < 1e-6] ['associationId'].tolist()

 >  >  > my_associations2 = my_associations [association_ids]

To check risk alleles and risk frequency.

 >  >  > my_associations2.strongest_risk_alleles [ ['riskAlleleName', 'riskFrequency']]

### Example 2: in conjunction with other Python tools

The data type of pandasGWAS is pandas.DataFrame, which is the foundation of data analysis in python. It can be easily combined with other analysis and visualization tools. This example will be used in conjunction with plotnine [7] to visualize data. plotnine is an Python implementation of ggplot2 [8], which is a grammar of graphics in R.

Firstly, we load the required modules in the Python console.

 >  >  > from pandasgwas.get_studies import get_studies

 >  >  > from plotnine import ggplot,geom_bar,aes

Secondly, we search Study based on different disease Trait. We can use the plus sign( +) to aggregate all results.

 >  >  > study1 = get_studies(reported_trait = 'Suicide risk')

 >  >  > study2 = get_studies(reported_trait = "Dupuytren's disease")

 >  >  > study3 = get_studies(reported_trait = "Triglycerides")

 >  >  > study4 = get_studies(reported_trait = "Retinal vascular caliber")

 >  >  > study5 = get_studies(reported_trait = "Non-small cell lung cancer (survival)")

 >  >  > all_studies = study1 + study2 + study3 + study4 + study5

**Fig. 5:**
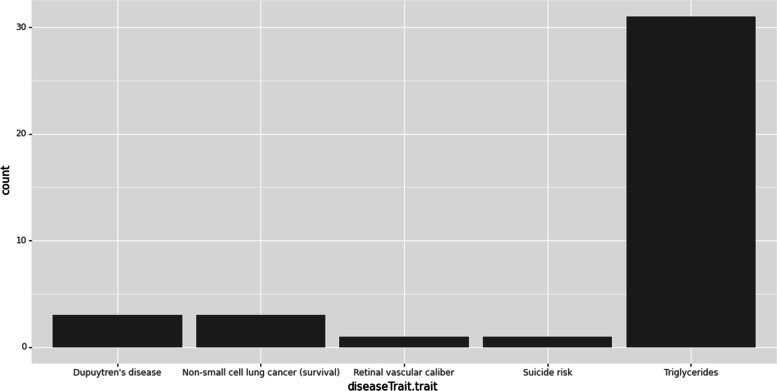
Analyze study by disease trait

In order to analyze the results of the query, we can also use the math symbol ( +) to complete the data visualization. From the graph, we know that the count of research related to "Triglycerides" is the most highest (Fig. 5).

 >  >  > ggplot(all_studies.studies) + geom_bar(aes(x = 'diseaseTrait.trait'))

### pandasGWAS vs gwasrappidd

Among the existing tools, gwasrappidd [9] which is implemented based on the R programming language is the only one with similar functionality to pandasGWAS. Users familiar with gwasrapidd can easily use pandasGWAS in Python. First, use "from pandasgwas import *" instead of "library(gwasrapidd)" in R to import the functions into current namespace. Second, the names of the functions starting with "get" and their main arguments in pandasGWAS are the same as in gwasrapidd. Users need to pay attention to the differences of types between R and Python when using parameters. For more detailed information about the types of functions in pandasGWAS, one can refer to the definitions and examples given in the GITHUB.IO documentations.

Compared with gwasrapidd, pandasGWAS has several advantages and we just list some of them below. The function set_xor() is not supported by gwasrapidd and it does not support mathematical symbol operations to simplify set operations on the requested data (Table 1). The mapping between the columns of the table and the keys of JSON in the GWAS Catalog REST API is weak. Firstly, some data are missing, such as: locations of Variant in API. Secondly, when some data is flattened and assigned to child DataFrames, gwasrappidd does not create primary and foreign keys to indicate the relationship between them, such as: variants.ensemble_ids in gwasrappidd. When researchers used gwasrappidd for the first time, confusions between the official website's REST API and the results returned by the function may be caused by the weak mapping. However, pandasGWAS can solve most of the problems mentioned above in gwasrappidd.

**Table 1:** pandasGWAS vs gwasrappidd

	pandasGWAS	gwasrappidd
Programming Language	python	R
Type of Property	pandas.DataFrame	tidyverse.tibble
Set Operations	set_xor, bind, union, intersect, set_diff, set_equal	bind, union, intersect, set_diff, set_equal
Set Operations Based on Mathematical Symbol	+ (bind), &(intersect), -(set_diff), ^(set_xor), |(union), = = (set_equal)	Unsupported
The Mapping Between The Columns of The Table and The Key of JSON in The GWAS Catalog REST API	Strong	Weak

## Conclusions

pandasGWAS definitely fills a major gap in the Python community for programmatic access to the GWAS Catalog data. Compared to existing tools, pandaGWAS is easier to get started. pandasGWAS is tested and documented, which has been uploaded to PyPI and can be easily installed by typing "pip install pandasgwas" at the command line.

## Availability and requirements

Project name: pandasGWAS.

Project home page: https://pypi.org/project/pandasgwas

Operating system(s): any supporting Python >  = 3.8 (tested on Windows 10).

Programming language: Python.

Other requirements: pandas >  = 1.4.3, requests >  = 2.28.1, progressbar2 >  = 4.0.0.

License: MIT License.

Any restrictions to use by non-academics: The NHGRI-EBI GWAS Catalog and all its contents are available under the general terms of use for EMBL-EBI services.

## Data Availability

Source code is available in https://pypi.org/project/pandasgwas and https://github.com/caotianze/pandasgwas. Documentation and tutorials can be found at https://caotianze.github.io/pandasgwas/.

## Abbreviations

GWAS: Genome-wide association studies
NHGRI: National Human Genome Research Institute
EBI: European Bioinformatics Institute
REST: Representational State Transfer
API: Application Programming Interface
JSON: JavaScript Object Notation
HAL: Hypertext Application Language
HTTP: Hyper Text Transfer Protocol
URL: Uniform Resource Locator
EFO: Experimental Factor Ontology
